# Brain size predicts bees' tolerance to urban environments

**DOI:** 10.1098/rsbl.2023.0296

**Published:** 2023-11-29

**Authors:** Jose B. Lanuza, Miguel Á. Collado, Ferran Sayol, Daniel Sol, Ignasi Bartomeus

**Affiliations:** ^1^ Estación Biológica de Doñana (EBD-CSIC), 41092 Seville, Spain; ^2^ Spatial Interaction Ecology, German Centre for Integrative Biodiversity Research (iDiv) Halle-Jena-Leipzig, Leipzig, Saxony, Germany; ^3^ Departamento de Ciencias de la Computación e Inteligencia Artificial, Universidad de Sevilla, Seville, Spain; ^4^ Centre for Ecological Research and Forestry Applications (CREAF), Bellaterra, Catalonia, Spain; ^5^ Department of Ecology, CSIC, Spanish National Research Council, CREAF-UAB, Bellaterra, Catalonia, Spain

**Keywords:** relative brain size, habitat occupancy, Apoidea, urbanization, pollinators

## Abstract

The rapid conversion of natural habitats to anthropogenic landscapes is threatening insect pollinators worldwide, raising concern regarding the negative consequences on their fundamental role as plant pollinators. However, not all pollinators are negatively affected by habitat conversion, as certain species find appropriate resources in anthropogenic landscapes to persist and proliferate. The reason why some species tolerate anthropogenic environments while most find them inhospitable remains poorly understood. The cognitive buffer hypothesis, widely supported in vertebrates but untested in insects, offers a potential explanation. This theory suggests that species with larger brains have enhanced behavioural plasticity, enabling them to confront and adapt to novel challenges. To investigate this hypothesis in insects, we measured brain size for 89 bee species, and evaluated their association with the degree of habitat occupancy. Our analyses revealed that bee species mainly found in urban habitats had larger brains relative to their body size than those that tend to occur in forested or agricultural habitats. Additionally, urban bees exhibited larger body sizes and, consequently, larger absolute brain sizes. Our results provide the first empirical support for the cognitive buffer hypothesis in invertebrates, suggesting that a large brain in bees could confer behavioural advantages to tolerate urban environments.

## 1. Introduction

Pollinators deliver a fundamental ecosystem service on which the Earth's vegetation and human economy depend [1]. Regrettably, there is increasing evidence of recent declines in pollinator populations [2–4]. One of the main contributing factors to the current pollinator declines is the alteration and loss of their habitat due to human activity [5,6]. Anthropogenic landscapes present new challenges for the survival and reproduction of organisms, increasing their risk of extinction by maladaptation [7–9]. Yet, not all pollinator species are negatively affected by land use change. Indeed, some bee species are able to tolerate human-altered environments [10–12] or even to thrive [13,14].

Human-dominated habitats, notably cities, drastically modify the ancestral conditions where pollinators evolved, but can also offer unique ecological opportunities in the form of new nesting spots, shelter from phytosanitary products, reduced predation pressure and high food availability associated with non-indigenous plants [7,12,15]. The question is then why are only some species able to tolerate and exploit urban environments. The ‘cognitive buffer hypothesis’ provides an explanation for this conundrum, suggesting that in novel environments the chances to successfully survive and reproduce depends on enhanced cognition to gather, store and plastically react to new information [16,17]. While the ‘cognitive buffer hypothesis’ receives ample support from studies of vertebrates [18–21], similar evidence is lacking for insects.

Insects have long been associated with the most basic types of learning due to their miniature brain [22,23]. However, recent studies have shown that bees and other insects have sophisticated cognition that goes beyond simple associative learning or conditioning [24–26]. Some of these more complex forms of cognition involve the use of tools, attention, social learning or metacognitive processes [22,23]. In addition, there is also evidence for substantial variation across species in brain size, both in absolute terms and relative to body size, with species that have larger brains also exhibiting enhanced cognitive performance, at least for some tasks such as conditional learning [27]. Therefore, we can ask whether the varying success of insects in human-altered habitats might be explained by variation in brain size.

Here, we report the first test of the ‘cognitive buffer hypothesis' in insects. Our test is based on a unique dataset of brain measures for 89 European and North American bee species. By means of detailed georeferenced information of species occurrences, we characterize the degree of habitat occupancy for all the species, and use a phylogenetically informed comparative analysis to assess whether bees that proliferate in human-altered habitats have enlarged brains compared to those that avoid them.

## 2. Methods

### (a) Brain measurements

Our dataset contains measurements of brain and body size for bee specimens captured on flowers by hand netting in different areas of the East Coast of the United States and northern Central Europe. These specimens were collected opportunistically mostly in semi-natural habitats. The dataset includes information of 335 female individuals from 89 species that represent six families and 31 genera. We considered only female specimens because they engage in a wider variety of tasks, facing greater environmental pressures, and possess distinct brain structures and functions compared to males [28,29]. Brain size was measured as the weight of fixed brains [30] and body size as intertegular span ([31]; electronic supplementary material, text S1). Given that brain size scales allometrically with body size [30], we considered for our analysis the size of the brain relative to the body.

**Figure 1.**
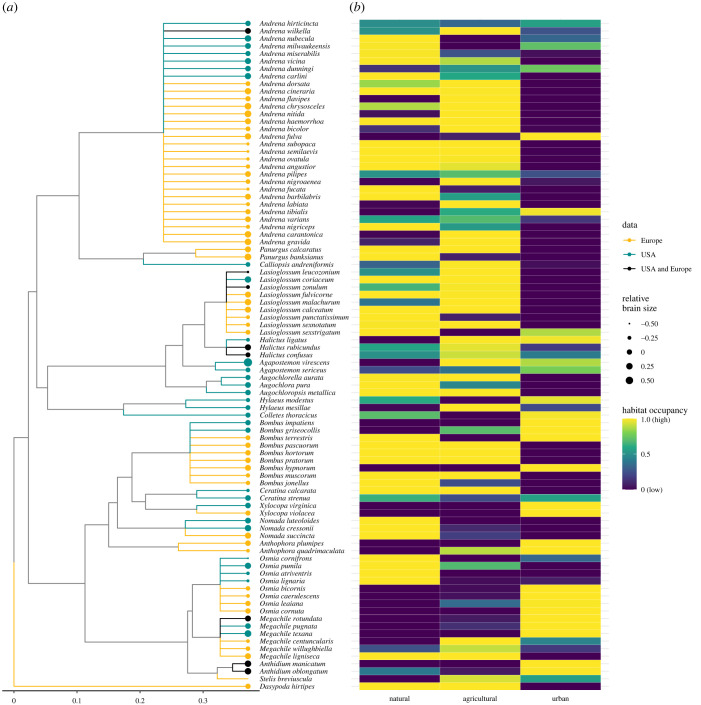
Phylogenetic relationship and degree of habitat occupancy for the selected bee species with brain weight and intertegular distance information (*N* = 89). (*a*) Phylogenetic tree at the genus level. Tree branches are coloured based on the geographical location of the different bee species (northern Central Europe, East Coast of the United States or from both regions). The deviation of the brain size in relation to the body (i.e. residuals) is represented with filled circles of proportional area at the end of the tip branches. Larger circles indicate larger brains in proportion to their body size and vice versa. (*b*) Heat map showing the degree of habitat occupancy for each bee species. The columns delimit the habitat type (i.e. natural, agricultural and urban) and the rows the different bee species.

Following Sayol *et al.* [30], we estimated relative brain size as the residuals of a log–log phylogenetic linear model of brain mass against body size, using the Bayesian approximation implemented in the function *brm* from the package *brms* v. 2.18 [32]. The phylogeny was built with the help of a previously published genus-level phylogeny [33] and it was processed with the help of the packages ape 5.6-2 [34], phytools 1.2-0 [35] and MCMCglmm v. 2.33 [36]. High values of relative brain size indicate larger brains than expected for their body size, while low values denote smaller brains. Our bee dataset showed a strong allometric relationship between brain and body size (Bayesian *R*^2^ = 0.9) that was constrained by the species’ evolutionary history (phylogenetic signal of relative brain size, *λ* = 0.6; *p* < 0.01). However, we found considerable variability in relative brain size within and across taxonomic groups (electronic supplementary material, figure S1; figure 1*a*).

### (b) Habitat occupancy

We downloaded occurrence information for all the measured species from the Global Biodiversity Information Facility (GBIF; http://www.gbif.org/) for North America and Europe. The data were downloaded via the R programming language with the help of the function *occ_download* from the package *rgbif* v. 3.7.3 [37]. We selected the states or countries with the highest density of records for our set of species. For North America, we selected states located on the East Coast of the United States (electronic supplementary material, figure S2*a*), covering an approximate area of 136 937 km^2^. For Europe, we selected countries located on the north and centre of the continent (electronic supplementary material, figure S2*b*), representing a total area of 600 497 km^2^. We only included species with a minimum number of 50 records and whose geographical distribution was larger than the sampled area (i.e. excluding species at the edge of their distributions). We optimized the match between species occurrence and the land cover data by only using georeferenced records obtained between 1990 and 2022 with a minimum of two decimals of latitude/longitude coordinates.

We assigned a habitat type to each GBIF occurrence by merging land cover information with the georeferenced records of species occurrences. The land cover classification was obtained from the 2006 online inventories of the National Land Cover Database (NLCD) for the United States and the Corine Land Cover (CLC) for Europe. After downloading these inventories as raster files, we used the functions *rast* and *extract* from the *Terra* package v. 1.6-41 [38] to read and obtain the cover classification of the different georeferenced records, respectively. To simplify the interpretation and conduct a joint analysis for both regions, we divided the resulting cover classes into three single categories: (i) natural, (ii) agricultural and (iii) urban (see electronic supplementary material, tables S1 and S2 for details). With this information we can build an occurrence matrix with species in rows, habitats in columns and cells depicting the number of occurrences per species–habitat combination.

The degree of habitat occupancy was estimated by assessing whether the occurrences of species in a given habitat were more frequent than expected by chance. Although previous work has referred to this metric as ‘habitat preference’ [12], we have opted to use ‘degree of habitat occupancy’ as it avoids the connotation of active choice by the different species. To calculate habitat occupancy, we generated 10 000 randomized matrices based on the occurrence matrix with the function *nullmodel* from the package *bipartite* v. 2.16 [39]. We used the method ‘r2dtable’, which maintains constant row and column sums by using Patefield's algorithm [40]. This maintains constant the proportional dominance of the species and habitats but reshuffles their associations. We then estimated the percentage of simulated occurrences per species and habitat that were under the observed ones (i.e. percentile). Lastly, we considered that a bee species exhibited a ‘high habitat occupancy’ for any of the three studied habitats when the number of occurrences observed in this habitat exceeded the 80th percentile of the values obtained from the simulations. On the contrary, we considered a ‘low habitat occupancy’ for the habitat when the observed occurrences were below the 20th percentile. To better understand if our findings are affected by the evolutionary history of the species, we estimated the phylogenetic signal of relative brain size and the degree of habitat occupancy across habitats for our set of species with the help of the function *phylosig* from the *phytools* package.

### (c) Diet specialization

To ensure diet is not acting as a confounding factor we investigated how diet specialization is associated with the degree of habitat occupancy and brain size. For this, we used existing diet information (see [30]), where bees were classified as oligolectic when they use a single plant family to feed their brood or polylectic when they use several. We tested for statistical differences in diet across the degree of habitat occupancy per habitat type and brain size by using Wilcoxon's test. We found a low number of specialist species in our dataset with no clear associations with the degree of habitat occupancy or brain size that can explain the observed relationship between the two (electronic supplementary material, figure S3).

### (d) Analysis

To evaluate how the association between the degree of habitat occupancy and relative brain size per species changed by habitat type, we used a phylogenetic Bayesian approach to model their association. For this, we first joined the resulting values of the degree of habitat occupancy per species with their respective average relative brain sizes. Our macro-ecological approach is justified by: (i) independent data ensuring robust and generalizable ecological patterns and (ii) minimal intraspecific variation in brain and body size compared to interspecific variation (see electronic supplementary material, text S2 and figure S4). The resulting distribution of the degree of habitat occupancy for each of the habitats analysed followed a 0–1 inflated beta distribution (electronic supplementary material, figure S5), indicating that there were high frequencies of habitat occupancy close to 0 or 1 but low frequencies of intermediate values between 0 and 1. In other words, simulated occurrences per species and habitat were consistently lower or higher than the observed ones. Hence, in our analyses we take a conservative approach and only modelled the extremes of the distribution (i.e. species classified as high or low habitat occupancy). Because we assessed the degree of habitat occupancy as binary (‘low’ or ‘high’), we specified a Bernouilli distribution where the degree of habitat occupancy was the response variable and relative brain size the predictor. To control for the shared evolutionary history of the species, we included the phylogenetic covariance matrix as a random factor. Finally, we replicated the analyses for the United States and Europe independently with analogous models.

All our models were run with 4000 iterations with previous 1000 warm-up iterations, using non-informative or weakly informative priors [32]. Further, the different posterior predictive checks were conducted with the function *pp_check*, also from the *brms* package. All our analyses were undertaken in R v. 4.0.5 [41], and all data processing and graphics were done with the set of packages from the tidyverse v. 1.3.0 [42].

## 3. Results

The degree of habitat occupancy varied substantially across species (figure 1*b*) and showed moderate phylogenetic signal *λ* = 0.38; *p* = 0.02). In general, most bee species have high occupancy in one or two habitat types but more rarely occurred indistinctly in the three (figure 1*b*). The habitats with the highest occurrences in comparison with random expectations were the agricultural and natural ones, with 49 and 48 species over 80% of the values from null models, respectively (figure 1*b* and electronic supplementary material, figure S5). By contrast, most species showed a low degree of occupancy in urban habitats (56 species under the 20th percentile) and just 28 species showed high occupancy in this habitat type (figure 1*b* and electronic supplementary material, figure S5).

**Figure 2.**
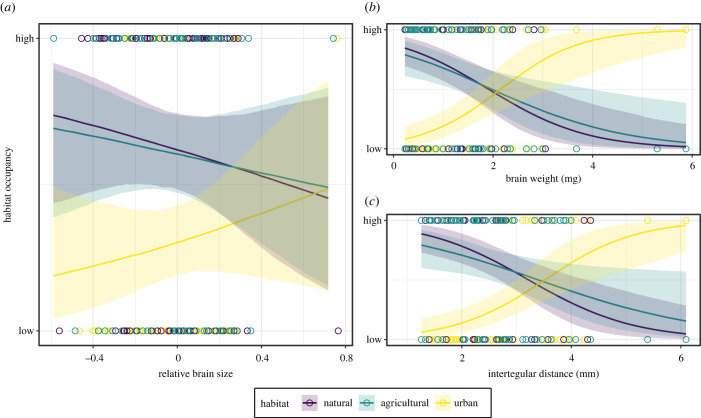
Association between relative brain size (*a*), brain weight (*b*) and intertegular distance (*c*) with the degree of habitat occupancy by habitat type (i.e. natural, agricultural and urban). The shaded and coloured areas by habitat type represent 95% credible intervals. Coloured circles indicate the raw values of relative brain size for the resulting levels of habitat occupancy.

We found that relative brain size was associated with the degree of occupancy across the different habitat types (figure 2*a*; Bayesian *R*^2^ = 0.11). Specifically, we found that bees with larger relative brains showed a higher occupancy in urban habitats than bees with smaller ones (figure 2*a*). Contrarily, bee species with smaller relative brains showed higher occupancy in natural and agricultural habitats than bees with larger relative brains (figure 2*a*). The models of the association between absolute brain size and intertegular span with habitat occupancy also showed a marked difference between habitat types (figure 2*b*,*c*; Bayesian *R*^2^ = 0.25; Bayesian *R*^2^ = 0.23, respectively). Specifically, we found that bees with larger brains and body sizes appear more in urban habitats and bees with smaller brains and body sizes are more common in natural and agricultural ones (figure 2*b*,*c*). These findings were consistent with the analogous analyses by geographical regions (United States and Europe; electronic supplementary material, figure S6).

## 4. Discussion

By analysing 89 bee species from two different continents, we observed that bee species inhabiting urban habitats tend to have larger brains relative to their body while those found in forest or agricultural habitats exhibit relatively smaller brains. These results are in line with the cognitive buffer hypothesis [17], which predicts that a large relative brain should provide enhanced behavioural plasticity to tolerate novel environments. Since urban environments exhibit dynamic challenges, including novel resources and changing human disturbances [43], a large brain may provide the cognitive flexibility to exploit these new resources while avoiding risks. Although the exact mechanisms remain unclear, we expect cognitive flexibility to be essential in a variety of contexts such as the use of human-made materials for the nest [44] or the use of exotic flowers [45].

In line with the cognitive buffer hypothesis [46], urban bees also showed bigger bodies and larger absolute brains. Larger species require longer development times and obtain greater net benefits from exploring and learning, especially in heterogeneous environments [47,48]. For example, the large carpenter bees of the genus *Xylocopa* can live up to 2 years and are frequent urban dwellers, while small *Andrena* forest specialists complete their adult life cycle in a few weeks. The patchiness of urban resources also seems to favour larger body sizes, as suggested by the observation that foraging distance is positively associated with body size in bees [31,49]. There is also some evidence that associate increased tolerance to urbanization with wider ecological niches [50–52], and both larger bodies and relative brains are thought to be features that can facilitate broader diets and niche expansion [20,53,54]. In our study, we examined only a few bee specialists, highlighting the need for further research on how different diets and life strategies relate to cognition and urban adaptation.

Our findings support and extend upon previous evidence in vertebrates that having larger brains can facilitate tolerance to urban environments [55,56], highlighting that a cognitive buffer is possible even with tiny brains. The use of the entire brain size as a proxy for cognitive performance is not exempt from criticism [24,57]. We primarily analysed brain size due to data availability, supported by prior findings showing larger brains improving certain cognitive aspects in bees, such as learning [27]. Moreover, brain size is less subject to measurement error or context-dependent biases in comparison with other experimental measures of cognition [24,57]. However, future finer measures, such as neuropil size or mushroom bodies (the suspected centres of cognitive processes in bees; [58,59,60]), are likely to enhance our understanding of bee cognition. Downscaling our analysis to the individual specimen level where brain size, habitat use and cognitive performance can be tracked through their lifespan would be a challenging but promising next step. Our findings highlight the importance of cognition for understanding the dynamics of insect populations in altered environments and stresses the need to avoid viewing them as passive agents of external pressures.

## Data Availability

Data available from the Dryad Digital Repository: https://doi.org/10.5061/dryad.zw3r228dr [61]. Electronic supplementary material is available online [62].
